# Peculiar conduction

**DOI:** 10.1007/s12471-025-01977-w

**Published:** 2025-08-27

**Authors:** Vladimir D. C. L’Espoir, Reinder Evertz, Rypko Beukema

**Affiliations:** Department of Cardiology, Radboud University Medical Centre, Nijmegen, The Netherlands

## Answer

**Fig. 1:**
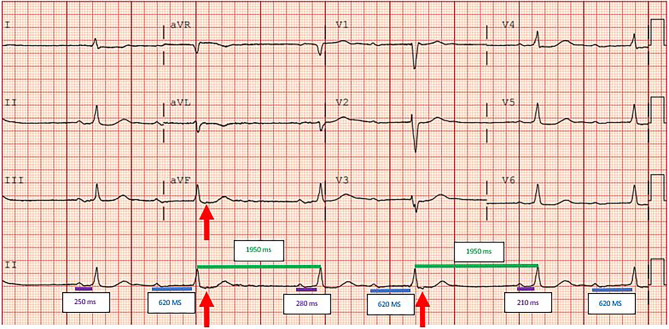
12-lead resting electrocardiogram performed at the outpatient clinic

The electrocardiogram (ECG) in Fig. 1, shows a sinus rhythm with a first-degree atrio-ventricular (AV) block, with a PQ interval of approximately 620 msec (highlighted by the blue line).

**Fig. 2:**
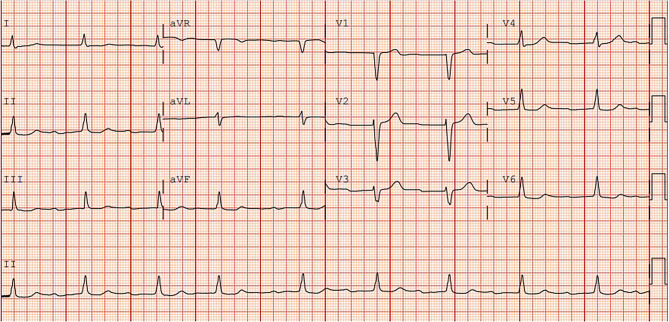
12-lead resting electrocardiogram performed at outpatient clinic 2023

The PQ intervals of the first, third and fifth heartbeats (the purple lines) are not equal, but vary between 210 and 280 msec. Furthermore, the patient has a known history of a first-degree AV block, as seen on the previous outpatient ECG (Fig. 2). Taken together, these findings suggest there is no consistent relation between these P waves and the following QRS complexes, indicating a lack of antegrade AV conduction during these cycles. However, the stable PQ interval indicated by the blue lines does show antegrade conduction, albeit with an extreme prolonged PQ interval. This is consistent with the prior outpatient ECG (Fig. 2), confirming our diagnosis of first-degree AV block.

The RR intervals between the second and third, as well as the fourth and fifth beats (green lines), are equal, suggesting a recurring relation—consistent with an escape interval. This is especially relevant in light of the previously mentioned variability in the purple PQ intervals and the known first-degree AV block, further supporting the absence of AV conduction between these P waves and these QRS complexes.

The irregularity of the PP intervals can be explained by resetting of the sinus node via rapid retrograde ventriculo-atrial (VA) conduction. Retrograde VA conduction may occur either via a concealed accessory pathway or through retrograde AV node conduction [1, 2]. This retrograde conduction is highlighted by the red arrows, which indicate retrograde P waves. Due to the extremely prolonged PQ interval in this case of first-degree AV block, the atria are non-refractory and thus receptive to VA conduction. Dhir et al. reported a prevalence of VA conduction in 11.9% of patients with AV block [1]. This retrograde P wavea resets the sinus node, resulting in a compensatory pause.

Eventually, the patient developed complete AV block and was treated with a DDD pacemaker. A pacemaker-mediated tachycardia was observed demonstrating the impact of retrograde VA conduction inappropriately triggered by ventricular pacing.
